# Effects of Pilates combined with breathing exercise on lung function, body posture and postural stability among female college students: A randomized controlled trial

**DOI:** 10.1371/journal.pone.0330874

**Published:** 2025-08-20

**Authors:** Jie Zhang, Yanan Zhao, Qianwen Wang

**Affiliations:** 1 College of Physical Education, China Three Gorges University, Yichang, Hubei, China; 2 School of Sports Science and Physical Education, Nanjing Normal University, Nanjing, Jiangsu, China; 3 The Department of Orthopedics & Traumatology, The Chinese University of Hong Kong, Hong Kong, China; Japanese Academy of Health and Practice, JAPAN

## Abstract

**Objective:**

This study aimed to explore the effects of Pilates combined with breathing exercise on lung function, body posture, and postural stability among female university students.

**Methods:**

A total of 66 females (mean age 19 years) with poor body posture were recruited from a local university and randomly divided into three groups, Pilates combined with breathing exercise group (PRT), Pilates only group (PLT), and control group (CON). Exercise interventions were conducted three times per week, 60 min per session, and lasted 16 weeks (8 weeks of group training + 8 weeks of self-training). Lung function and respiratory muscle performance, as the primary outcomes were measured using the Lung Function Tester. Secondary outcomes were standing posture and static postural stability.

**Results:**

Significant group differences were found at post-test in Forced Vital Capacity (FVC) (*F*(2, 50) = 3.63, **p* *= 0.034, p*η*^*2*^* *= 0.13) and Minute Ventilation (MV) (*F*(2, 50) = 3.52, **p* *= 0.04, p*η*^*2*^* *= 0.123), where the PRT group showed more improvements than the PLT group especially in FVC (mean difference = 0.43, **p* *< 0.05). Furthermore, the PRT group showed significant improvements at post-test in Forced Expiratory Volume in 1 second as a percentage of Forced Vital Capacity (FEV1%) (*F*(2, 42) = 10.2, **p* *< 0.01, p*η*^*2*^* *= 0.327), Peak Expiratory Flow Rate (PEFR) (*F*(2, 42) = 5.62, **p* *= 0.01, p*η*^*2*^* *= 0.211) and Tidal Volume (TV) (*F*(2, 42) = 8.38, **p* *< 0.001, p*η*^*2*^* *= 0.285). Additionally, it improved body posture and static postural stability, with notable gains in certain stability measures under both eyes-open and eyes-closed conditions (**p* *< 0.05).

**Conclusions:**

Combining breathing exercises with Pilates can improve lung function, body posture, and postural stability in female college students, and a longer training duration (> 16 weeks) appears beneficial for achieving optimal outcomes. These findings suggest a potential association between lung function and postural stability mediated by respiratory muscle function, which warrants further investigation.

## Introduction

Due to the widespread use of the internet and electronic devices worldwide, people tend to lead sedentary lifestyles [1]. Approximately 23% of adults and 81% of adolescents worldwide are physically inactive [2]. Among college students, female students have significantly lower levels of physical activity than their male counterparts [3]. Insufficient engagement daily physical activity has been associated with an increased risk of chronic diseases and reduced mental well-being. Furthermore, various spinal problems have been reported in college students due to their poor posture, such as forward head posture, rounded shoulders, scoliosis, and anterior pelvic tilt [4]. Poor body posture can reduce activation of deep core muscles such as the transversus abdominis and multifidus, thereby restricting diaphragmatic descent during respiration. This impaired coordination may decrease thoracic mobility, limiting the effective expansion of the lungs, especially during deep breathing [5]. To ensure sufficient oxygen reserves for metabolism, individuals with poor posture often compensate by activating accessory respiratory muscles, such as the scalenes, sternocleidomastoid, and thoracic muscle groups [6,7]. However, this compensatory activation may restrict thoracic expansion and ultimately compromise respiration efficiency.

Respiratory performance largely depends on the respiratory pattern, which imposes varying levels of load on the primary and secondary respiratory muscles [8]. Additionally, it is influenced by the coordination of respiratory muscles [9]. The diaphragm also contributes to postural stability by regulating intra-abdominal pressure [10]. Inefficient breathing patterns can lead to muscular imbalances, which in turn contribute to poor body posture. Poor body posture further reduces respiratory efficiency by limiting thoracic expansion and respiratory muscle function. As a result pulmonary ventilation per unit of time is reduced [11]. This effect is particularly evident in sedentary posture, where respiratory flow rate is restricted compared to a normal upright posture [12]. In contrast, deep breathing patterns such as abdominal breathing enhance not only pulmonary ventilation but also the expansion and contraction capacity of the respiratory muscles [13]. Potential underlying mechanisms may exist between respiratory function and body posture.

Regular exercise has been positively associated with respiratory health and the development of postural muscle groups [14]. Both physical activity and specific respiratory muscle training have been shown to effectively improve dyspnea in patients with chronic obstructive pulmonary disease [15,16]. Pilates is an integrated exercise method that focuses on strength, core stability, flexibility, muscle control, posture, and breathing [17,18]. This method engages the deep muscle groups that maintain and coordinate body posture, thereby supporting and protecting the spine. The deep core muscles, including the transverse abdominis, multifidus, pelvic floor, and diaphragm, play a key role in providing alignment and posture support. In addition, enhancing the function of these deep core muscle groups helps activate intrinsic stabilizing muscles of the abdomen, lower back, hips, and spine, thereby balancing back and abdominal muscle strength and correcting poor body posture [18]. While most existing studies have focused on clinical populations such as individuals with COPD or other chronic conditions, there is still limited evidence regarding exercise interventions for healthy individuals with poor posture. Furthermore, these studies are often no longer than eight weeks in duration and lack long-term follow-up data [19,20]. Notably, few studies have investigated whether improvements in pulmonary function are mediated by the activation of postural and respiratory stabilizing muscles. This study aimed to examine the effects of exercise on lung function, body posture and postural stability among female college students with poor body posture and the linking effects of an exercise intervention on respiratory and postural stabilizing muscle groups.

## Method

### Study design

This randomized, single-blinded, parallel-controlled, three-group study was conducted following the Consolidated Standards of Reporting Trials statement [21]. In accordance with the Declaration of Helsinki. This study was approved by the Ethics Committee of Nanjing Normal University (No. 202007018), and prospectively registered in the Chinese Clinical Trial Registry (ChiCTR2000032345).

### Sample size estimation

Previous studies commonly reported moderate effect sizes (Cohen’s *d* ≈ 0.4–0.5) for exercise interventions targeting lung function parameters [22,23]. Based on these findings, an effect size of 0.4 was selected for sample size estimation. A priori power analysis was performed using G*Power (version 3.1.9.7), employing an ANCOVA model with covariate. The analysis indicated that 52 participants were required. Accounting for a possible 10% dropout rate and additional variability, a total of 66 participants (22 per group) were recruited.

### Grouping and randomization

Consenting participants were coded and entered into a computer system, then randomly assigned to three equal groups based on a computer-generated sequence. Group allocation was performed by a student assistant who was not involved in any other research procedures. Participants were randomly assigned to an experimental intervention group (PRT: Pilates combined with breathing exercise), an active control group (PLT: Pilates only), and a blank control group (CON).

### Participant

**Fig 1:**
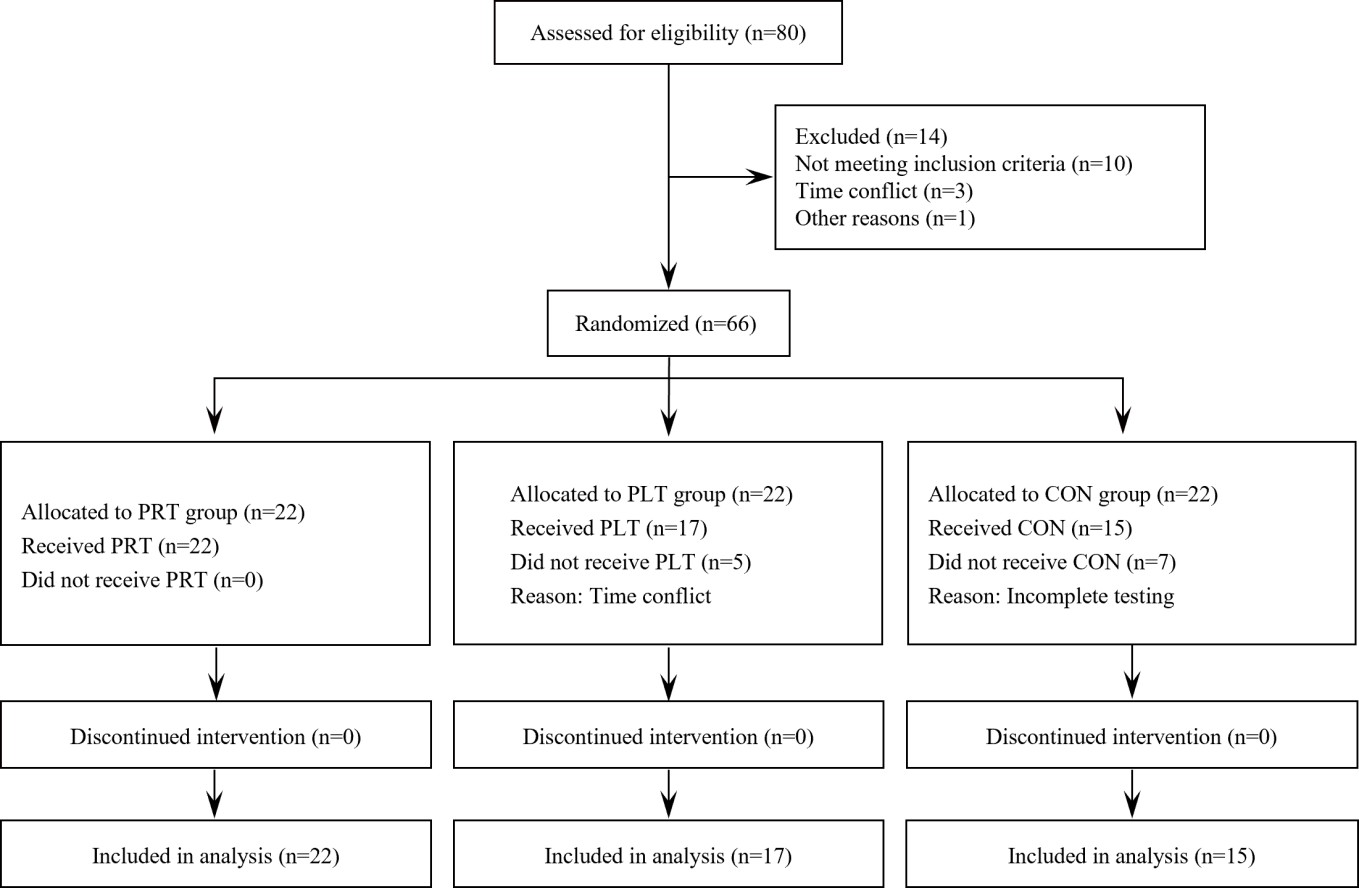
Flowchart of the study. This figure shows the sequence of experimental steps including recruitment, intervention, and analysis.

Participants were recruited for observation between 15 April and 1 September 2020. A total of 80 female college students aged between 18 and 22 years from a local university were recruited through posters and lectures promoting a healthy lifestyle. However, 14 participants were excluded due to not meeting the inclusion criteria, scheduling conflict with the intervention, or other reasons. The study flowchart is presented in Fig 1.

The inclusion criteria were as follows: 1) prolonged sedentary study or work; 2) no regular daily exercise habits; and 3) at least one poor body posture type, such as: rounded shoulders (typically characterized by protracted and internally rotated shoulders, and sometimes accompanied by scapular elevation), forward head posture (forward head angle < 48°), or anterior pelvic tilt (anterior pelvic tilt angle > 15°). Participants were excluded if they displayed any physical unfitness for exercise or time conflict with intervention. Additionally, all participants confirmed having no prior experience with Pilates training and provided written informed consent prior to enrollment. Data collection was carried out in the fitness testing laboratory.

### Intervention

#### Training program design.

According to *Pilates*, the book written by Joseph Hubertus Pilates, the founder of the method [24], 24 typical Pilates movements were selected and included in the exercise intervention. The selected movements include neck and shoulder stretches, arm circles, supine marching, four-legged swimming, curl-ups, hundred taps, plank, chest lifts, single-leg circles, quadriceps stretches, opposite arm and leg reaches, supine spinal rotations, prone props, prone swimming, swan, V-spins, con-con dance, double leg extensions, mermaid side bends, side lying bicycle, shoulder bridge, pelvic curl, criss-cross, and kneeling side kick.

The 16-week exercise program was divided into four stages, with the quality of movements and exercise intensity were gradually increasing throughout the training process. Training for the PRT group was conducted from 16:00–18:00 on Monday, Wednesday and Friday, while training for the PLT group was carried out during the same time hours on Tuesday, Thursday and Saturday. A certified Pilates instructor supervised both groups in the same gymnasium to ensure consistent training guidance.

In the PRT group, participants were required to use a manual incentive spirometer to perform breathing exercises immediately after the Pilates training. They are instructed to breathe slowly and forcefully to maximize inhalation and exhalation depth. Upon reaching full inhalation capacity, the breath was held for at least ten seconds or as long as tolerable. After the ball reached the bottom of the column, participants paused briefly and repeated the process at least 10 times. A break was taken if dizziness or light-headedness occurred. The incentive spirometer is a mechanical hand-held breathing device in which the participant is instructed to take slow deep breaths through the device mouthpiece [25]. It provides participants with visual feedback on inhalation volume. A standard flow-oriented incentive spirometer consists of three chambers arranged in a row, each containing a colored ball. The flow threshold required to raise each ball is printed on the outside of its chamber. With an airflow rate of 600–1200 milliliters (mL) per second, the deep breath lifts the balls. When all three balls reach the top of the unit, the participant is considered to have achieved an inspiratory flow rate of 1200 mL per second. These colored balls offer a visual indication of breathing performance, which effectively improves adherence to slow, sustained deep inspiration.

Meanwhile, the PLT group participated exclusively in Pilates training, whereas the CON group received no exercise intervention and maintained their usual lifestyle throughout the study period. The entire intervention lasted 16 weeks. The first eight weeks involved supervised exercise at a gym, followed by eight weeks (weeks 9–16) of home-based online training. These home-based sessions were conducted live via Tencent Meeting at fixed times (Monday, Wednesday, and Friday afternoons). A single instructor led all sessions, providing real-time supervision, monitoring attendance through name-based check-ins, and observing participants via webcam to promote adherence and ensure consistent execution of the exercises.

#### Implementation of the training program.

**Fig 2:**
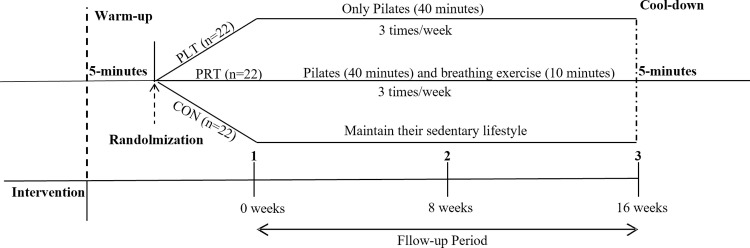
Study procedure. The complete intervention process, including warm-up, intervention, and cool-down phases performed by the participants.

Participants completed a 16-week intervention program, with three 60-minute sessions per week. Each training session comprised four phases: a i) 5-minute warm-up, ii) 40-minute Pilates training, iii) 10-minute breathing exercises (PRT), and iv) 5-minute cool-down. At the end of each phase, self-perceived exercise intensity was assessed using the Chinese version of the Rating of Perceived Exertion (RPE) scale [26]. The exercise intensities ranged from light (RPE 11) to somewhat hard (RPE 13). The detailed structure of the exercise implementation is presented in Table 1. Exercise intensity was monitored using heart rate and recorded before, during, and after each training session. Outcome indicators were collected at 0 weeks, 8^th^ week, and immediately after 16^th^ week. The entire study procedure is illustrated in Fig 2.

**Table 1:** Protocol of exercise intervention.

Pilates training	Week	Intensity (HRmax)	Load
Part I	1-4 weeks	55%	4-6/times * 3–4 group
Part II	5-8 weeks	60%	5-7/times * 3–4 group
Part III	9-12 weeks	65%	6-9/times * 3–4 group
Part IV	13-16 weeks	70%	8-11/times * 3–4 group

### Outcome measures

Pulmonary function measurements: Lung volumes and capacities were measured using a MiniSpir spirometer (MIR, Italy). Respiratory function was measured using the Lung Function Tester (AS-507, Japan) measuring forced vital capacity (FVC), forced expiratory volume in the first second (FEV1), FEV1/FVC (FEV1%), peak expiratory flow rate (PEFR), maximum voluntary ventilation (MVV), minute ventilation (MV), and tidal volume (TV). The respiratory function test was performed three times, and the highest value was recorded. PEFR and MVV were used to assess respiratory muscle performance, while FVC, FEV1%, MV, and TV were used to evaluate lung function. Participants were instructed to sit quietly for 10 minutes before the test to avoid any potential respiratory effects.

The static standing posture was assessed using photogrammetry [27]. Participants were asked to stand in a relaxed and natural position, facing an imaginary point on the opposite wall. Red adhesive markers were placed on the seventh cervical spinous process, the acromion and the tragus for postural analysis. Yellow adhesive markers were placed on the anterior and posterior superior iliac spines (ASIS and PSIS, respectively) to identify pelvic alignment during postural analysis. Participants stood side-on to the posture assessment chart with their left shoulder against the wall. The photographic tripod supporting the digital camera was positioned two meters from the assessment chart, aligned with the participant’s right shoulder level. Lateral photographs were taken, and the round shoulder angle (RSA) and the anterior pelvic tilt angle (APA) were calculated in the standing position of the participant using Photoshop. Bi-acromial breadth (BAD) and anterior pelvic tilt dimension (APD) were manually assessed using a flexible tape measure. For BAD, the tape was held parallel to the frontal axis of the body and extended between the left and right acromial points. The APD was measured at a point 2 cm below the participant’s navel.

Static postural stability was tested using the Footscan platform system (V.7.7, RSscan International, Olen, Belgium). The system was calibrated by measuring each participant’s weight and foot size before the test. Participants were instructed to stand on the force plate with the dominant leg, placing the instep of the non-dominant foot against the knee of the supporting leg. The participant’s upper limbs naturally hung at the sides of the body. Each test consisted of two 1-minute trials: one with eyes open and one with eyes closed. Participants whose feet touched the ground during the test were asked to rest for 30 seconds repeating the trial. The following data were recorded: maximum X-axis displacement (MS-X, mm), maximum Y-axis displacement (MS-Y, mm), X- and Y-axis displacement differences (X-Diff and Y-Diff, mm), center of pressure trajectory (COP, mm), and elliptical area (EA, mm^2^). All data were collected in the physiological laboratory on the ground floor of the College of Sports Science at Nanjing Normal University. Two research assistants who were blinded to group assignment and had received standardized training conducted all outcome assessments.

### Statistical methods

Demographic and clinical data are presented as means and standard deviations or as numbers and percentages. Data were analysed using the intent-to-treat method, and a sensitivity test was subsequently conducted using the available data. Missing values were imputed using the last-observation carried forward method. To compare differences in outcome changes between groups at mid- and post-intervention, a one-way analysis of covariance (ANCOVA) was conducted, with baseline values included as covariates. To assess within-group changes over time, a repeated measures analysis of variance (ANOVA) was applied separately for each group across the three time points (pre-, mid-, and post-intervention). Whenever violations arose in the sphericity assumption, the Greenhousee Geisser or HuynheFeldt correction was applied if ε < 0.75 or ε > 0.75, respectively [28]. Bonferroni corrections were applied to pairwise comparisons to control for type I error. All data were analysed using IBM SPSS Statistics V.26 (IBM Corp). Statistical significance was defined as p < 0.05.

## Results

### Participant flow

A total of 80 eligible female undergraduate students were recruited. More than 90% of the participants showed postural issues while standing, such as rounded shoulders and anterior pelvic tilt. Additionally, over 90% of the participants exhibited poor postural patterns during walking and sitting, such as body tilt, abnormal arm swing, foot dragging, elevated shoulders and thoracic kyphosis. Participants attended an average of 22 out of 24 in-person sessions (91.7%) and 21 out of 24 home-based sessions (87.5%). Table 2 shows the demographic and clinical characteristics of participants in each group. No significant differences were observed in baseline scores on the International Physical Activity Questionnaire (IPAQ) among the three groups (p > 0.05). Furthermore, no adverse effects were reported during the intervention period.

**Table 2:** Characteristics of participants.

Parameters	PRT (n = 22)	PLT (n = 17)	CON (n = 15)	All (n = 54)
**Age (years)**	19 ± 0.75	19 ± 0.70	19 ± 0.51	18.9 ± 0.69
**Height (cm)**	159.7 ± 6.3	159.7 ± 5.3	163.9 ± 5.2	160.9 ± 5.9
**Body Mass Index (kg·m**^**-2**^)	21.49 ± 3.02	20.34 ± 1.67	21.04 ± 2.47	21.0 ± 2.52
**Bust Circumference (cm)**	84.93 ± 6.46	82.10 ± 4.13	82.89 ± 5.29	83.47 ± 5.54
**Waist Circumference (cm)**	70.59 ± 8.04	68.43 ± 4.69	68.63 ± 3.73	69.36 ± 6.08
**Hip circumference (cm)**	92.81 ± 7.96	92.6 ± 2.33	94.5 ± 5.52	93.2 ± 5.95
**Systolic blood pressure (mmHg)**	103.2 ± 10.87	103.8 ± 13.94	114.3 ± 11.02	106.4 ± 12.7
**Diastolic blood pressure (mmHg)**	61.1 ± 9.55	60.9 ± 11.43	68.5 ± 10.38	63.05 ± 10.77
**Resting heart rate (beats/min)**	86.8 ± 16.47	87.28 ± 14.91	90.5 ± 12.06	88 ± 14.68
**IPAQ Score (MET-min/week)**	1678 ± 1245.6	1622 ± 1099.5	1607 ± 701.3	1635.7 ± 1041
**Types of pulmonary ventilation defects**				
**Normal pulmonary ventilation**	14 (63.6%)	12 (70.6%)	10 (66.7%)	36 (66.7%)
**Pulmonary ventilation disorders**	7 (31.8%)	4 (24%)	4 (27%)	15 (27.8%)
**Pulmonary ventilation limitation**	1 (4.5%)	1 (5.9%)	1 (6.7%)	3 (5.6%)

Normal lung ventilation (VC% ≥ 80%, FEV1.0% ≥ 80%); Lung ventilation disorders (VC% ≥ 80%, FEV1.0% < 70%); Lung ventilation limitation (VC% < 80%, FEV1.0 ≥ 70%); Mixed ventilation disorders (VC% < 80%, FEV1.0% < 70%); PRT = Pilates combined with breathing exercise; PLT = Pilates training; CON = Control group. IPAQ, International Physical Activity Questionnaire.

### Effects of time and group on lung function

Using the baseline data as the covariate (Table 3), ANCOVA results demonstrated significant group differences in FVC (*F*(2, 50) = 3.63, **p* *= 0.034, p*η*^*2*^* *= 0.13) and MV (*F*(2, 50) = 3.52, **p* *= 0.04, p*η*^*2*^* *= 0.123) at posttest. Planned contrasts revealed that the PRT group showed significantly greater improvement than the PLT group (difference = 0.43, p < 0.05). At mid-test, significant group differences were observed in MV (*F*(2,50) = 4.30, **p* *= 0.02, p*η*^*2*^* *= 0.147), with the PRT group performing significantly better than the PLT group (difference = 5.4, *p* = 0.02) and the CON group (difference = 1.5, *p* > 0.05). One-way repeated measures ANOVA showed a significant time effect on FEV1% (*F*(2, 42) = 10.2, **p* *< 0.01, p*η*^*2*^* *= 0.327) in the PRT group. Pairwise comparisons revealed a continuous increase from pre-test to mid-test (difference = −8.31, *p* < 0.01) and the posttest (difference = −9.02, *p* = 0.001). Results also revealed significant time effects in PEFR (*F*(2,42) = 5.62, **p* *= 0.01, p*η*^*2*^* *= 0.211) in the PRT group, where a continuous increase was observed from pre to-post-test (difference = −0.70, *p* = 0.02). In addition, significant time effects were observed in TV in the PRT group (*F*(2, 42) = 8.38, **p* *< 0.01, p*η*^*2*^* *= 0.285). Pairwise comparisons revealed a continuous increase from pre-test to mid-test (difference = −0.39, *p* < 0.005), and from pretest to posttest (difference = −0.33, *p* = 0.04).

**Table 3:** Within-group and between-group comparisons in lung function.

Tests	PRT			PLT			CON		
Lung function	Pre-test	Mid-test	Post-test	Pre-test	Mid-test	Post-test	Pre-test	Mid-test	Post-test
**FVC (L)**	2.86 ± 0.50	2.68 ± 0.61	2.95 ± 0.7	2.57 ± 0.48	2.49 ± 0.46	2.36 ± 0.27^*^	2.98 ± 0.39	2.83 ± 0.59	2.76 ± 0.54
**FEV1 (%)**	83.1 ± 14.4	91.4 ± 8.87 ^#^	92.2 ± 12.5 ^#^	84.7 ± 17.1	90.5 ± 8.13	95.4 ± 6.08	80.1 ± 22.5	87.6 ± 11.9	89.7 ± 12.3
**PEFR (%)**	3.34 ± 1.15	3.72 ± 0.90	4.04 ± 1.05 ^#^	3.23 ± 1.13	3.28 ± 0.81	3.71 ± 0.72	3.10 ± 1.08	3.69 ± 1.12	3.66 ± 1.20
**MVV (L/min)**	68.0 ± 21.2	67.8 ± 15.9	70 ± 19.8	60.4 ± 14.4	55.5 ± 16.7	57.5 ± 12.8	63 ± 19.8	62.9 ± 18.8	57.9 ± 20.2
**MV (L/min)**	15.5 ± 6.78	17.5 ± 8.36	18.1 ± 6.98	19.9 ± 9.41	14.9 ± 6.56 ^*^	15.9 ± 7.35 ^*^	11.9 ± 5.85	13.7 ± 6.62	11.8 ± 6.73
**TV (L)**	1.53 ± 0.50	1.92 ± 0.5 ^#^	1.86 ± 0.60 ^#^	1.57 ± 0.55	1.66 ± 0.40	1.59 ± 0.46	1.91 ± 0.49	1.88 ± 0.60	1.73 ± 0.71

FVC, forced vital capacity. FEV1%, FEV1/FVC. PEFR, peak expiratory flow rate. MVV, maximum voluntary ventilation. MV, minute ventilation. TV, tidal volume. PRT = Pilates combined with breathing exercise. PLT = Pilates training. CON = Control group.

* p < 0.05 vs. PRT at mid-test or post-test.

# p < 0.05 vs. pre-test.

### Effects of time and group in body posture

Based on the ANCOVA results, there were no significant differences in body posture-related parameters among the three groups. However, repeated measures ANOVA revealed significant time effects in RSA in both the PRT (*F*(2, 42) = 8.13, **p* *< 0.001, p*η*^*2*^* *= 0.279) and PLT groups (*F*(1.3, 20) = 4.21, **p* *= 0.046, p*η*^*2*^* *= 0.208; Table 4). Pairwise comparisons indicated significant decreases in RSA from pre-test to mid-test (PRT: difference = −11.45, **p* *= 0.01; PLT: difference = −11.62, **p* *< 0.001) and from pre-test to post-test (PRT: difference = −13.92, **p* *< 0.001). Significant time effects were also observed in APA in the PRT group (*F*(2, 42) = 8.29, **p* *= 0.001, p*η*^*2*^* *= 0.283), with a significant decrease in APA measured at post-intervention (difference = −4.45, **p* *= 0.002). Significant decreases in APD were observed in both the PRT group (*F*(2, 42) = 7.08, **p* *= 0.002, p*η*^*2*^* *= 0.252) and PLT groups (*F*(2, 32) = 5.14, **p* *= 0.012, p*η*^*2*^* *= 0.243). However, this time effect was only significant at the post-test (PRT: difference = 4.45, **p* *= 0.006; PLT: difference = 3.38, **p* *= 0.01). Significant time effects were also observed in BAD in both the PRT (*F*(2,42) = 1.184, **p* *= 0.000, p*η*^*2*^* *= 0.867) and PLT groups (*F*(2,32) = 11.48, **p* *= 0.000, p*η*^*2*^* *= 0.418). However, pairwise comparisons revealed significant decreases between pre-test and mid-test (PRT: difference = 2.36, **p* *< 0.001; PLT: difference = 1.35, **p* *= 0.01).

**Table 4:** Within-group and between-group comparisons in body posture.

Tests	PRT			PLT			CON		
Body posture	Pre-test	Mid-test	Post-test	Pre-test	Mid-test	Post-test	Pre-test	Mid-test	Post-test
**RSA (°)**	48.0 ± 12.9	59.4 ± 16.1^#^	61.9 ± 10.0^#^	52.5 ± 9.85	64.1 ± 6.59^#^	56.0 ± 16.73	50.8 ± 12.5	56.1 ± 12.6	55.1 ± 12.9
**APA (°)**	22.8 ± 5.65	20.1 ± 6.34	18.4 ± 5.84^#^	23.4 ± 5.79	21.2 ± 4.88	19.7 ± 4.88	22.4 ± 6.83	20.8 ± 5.88	17.5 ± 5.42
**APD (cm)**	84.0 ± 6.19	81.5 ± 7.83	79.5 ± 7.06^#^	82.3 ± 4.04	80.5 ± 5.05	78.9 ± 4.99^#^	82.9 ± 5.26	83.1 ± 7.62	80.9 ± 6.27
**BAD (cm)**	39.6 ± 1.51	37.2 ± 1.70^#^	40.2 ± 11.97	39.6 ± 1.43	38.3 ± 1.63^#^	38.1 ± 1.47	39.0 ± 1.83	39.1 ± 1.34	40.6 ± 6.75

RSA, round shoulder angle. APA, anterior pelvic tilt angle. APD, anterior pelvic tilt dimension. BAD, bi-acromial distance. PRT = Pilates combined with breathing exercise. PLT = Pilates training. CON = Control group.

# p < 0.05 vs. pre-test.

### Effects of time and group in static posture stability

ANCOVA results revealed significant group effects in static postural stability under eyes-closed conditions (Table 5). Significant group differences in COP were observed at post-test (*F*(2, 50) = 3.92, **p* *= 0.026, p*η*^*2*^* *= 0.136). Planned contrasts revealed that the PRT group showed significantly greater improvements than the PLT group (difference = 53.92, **p* *= 0.07), and the PLT group also showed significantly greater improvements than the CON group (difference = −65.49, **p* *= 0.04). After the intervention, significant group differences were revealed in Y-Diff (*F*(2, 50) = 3.81, **p* *= 0.03, p*η*^*2*^* *= 0.132), with the PLT group showing significantly higher performance than the CON group (mean difference = −40.29, **p* *= 0.03). Significant group differences were revealed in MS-Y at mid-test (*F*(2, 50) = 4.64, **p* *= 0.01, p*η*^*2*^* *= 0.157), with the PRT group showing significantly higher performance than the PLT group (difference = 18.44, **p* *= 0.02), and the PLT group showing greater improvements than the CON group (difference = −17.60, **p* *= 0.05).

**Table 5:** Within-group and between-group comparisons in static posture stability.

Tests	PRT			PLT			CON		
Postural stability	Pre-test	Mid-test	Post-test	Pre-test	Mid-test	Post-test	Pre-test	Mid-test	Post-test
**Eyes open**									
**MS-X (mm)**	299 ± 70.6	232 ± 79.2^#^	248 ± 91.9	281 ± 84.3	248 ± 80.1	232 ± 71.5	243 ± 65.3	252 ± 72.8	268 ± 58.9
**X-Diff (mm)**	51.68 ± 36.96	15.27 ± 8.30^#^	18.33 ± 8.47^#^	35.34 ± 30.89	13.30 ± 4.87^#^	14.87 ± 5.44^#^	23.33 ± 19.0	16.95 ± 7.43	20.45 ± 19.77
**MS-Y (mm)**	180 ± 24.6	178 ± 19.4	157 ± 30.2	172 ± 20.8	158 ± 41.6	162 ± 20.2	181 ± 19.1	179 ± 16.6	175 ± 21.4
**Y-Diff (mm)**	19.43 ± 8.54	15.06 ± 8.49	17.69 ± 5.10	22.38 ± 15.44	10.67 ± 2.39^#^	15.29 ± 6.96	15.86 ± 4.42	17.05 ± 11.23	15.44 ± 8.80
**EA (mm**^**2**^)	9.43 ± 4.26	9.54 ± 5.10	10.6 ± 4.91	9.30 ± 4.21	9.30 ± 4.21	8.27 ± 5.51	8.49 ± 3.20	10.57 ± 5.22	9.57 ± 4.61
**COP (mm)**	53.71 ± 22.66	56.12 ± 29.0	55.4 ± 18.29	62.08 ± 35.43	41.91 ± 13.53	44.15 ± 24.48	55.69 ± 26.07	52.20 ± 26.97	52.95 ± 25.56
**Eyes closed**									
**MS-X (mm)**	315 ± 62.5	249 ± 60.4^#^	269 ± 81.9	316 ± 63.4	265 ± 76.6^#^	250 ± 72.3	291 ± 62.9	266 ± 54.7	280 ± 68.7
**X-Diff (mm)**	105.38 ± 50.83	58.50 ± 34.04^#^	58.17 ± 37.46^#^	97.74 ± 55.86	50.76 ± 32.88^#^	39.52 ± 21.02^#^	96.51 ± 53.50	59.17 ± 29.01^#^	76.46 ± 79.23
**MS-Y (mm)**	190 ± 24.6	192 ± 21.6	172 ± 38.8	195 ± 24.2	175 ± 18.2^*^	172 ± 28.0	188 ± 33.1	191 ± 21.0^†^	200 ± 39.4
**Y-Diff (mm)**	67.56 ± 44.42	47.28 ± 22.86	42.51 ± 16.78^#^	57.55 ± 33.92	39.63 ± 26.12	31.62 ± 18.12^#^	63.02 ± 30.07	48.67 ± 27.10	72.27 ± 75.77^†^
**EA (mm**^**2**^)	20.98 ± 36.54	12.22 ± 6.51	12.57 ± 8.82	15.26 ± 7.41	13.16 ± 6.08	15.97 ± 8.76	16.17 ± 8.41	15.10 ± 8.31	32.08 ± 80.89
**COP (mm)**	201 ± 168.3	156 ± 74.4	162 ± 74.0	166 ± 94.5	142 ± 110.8	102 ± 35.1^*^	257 ± 144.6	182 ± 118.7	184 ± 103.2^†^

MS-X, maximum X-axis displacement. X (Y)-Diff, X- and Y-axis displacement differences. MS-Y, maximum Y-axis displacement. EA, elliptical area. COP, center of pressure. PRT = Pilates combined with breathing exercise. PLT = Pilates training. CON = Control group.

* p < 0.05 vs. PRT at mid-test or post-test.

# p < 0.05 vs. pre-test.

† p < 0.05 vs. PLT at mid-test or post-test.

Repeated measures ANOVA revealed significant time effects in X-Diff with eyes opened in both the PRT (*F*(1.14, 24) = 18.88, **p* *= 0.00, p*η*^*2*^* *= 0.473) and PLT groups (*F*(1.06, 17) = 7.50, **p* *= 0.01, p*η*^*2*^* *= 0.319). Pairwise comparisons indicated significant decreases from pre-test to post-test in both the PRT (difference = 33.4, **p* *= 0.001) and PLT groups (difference = 20.48, **p* *= 0.05). Significant time effects were also observed for MS-X in the PRT group with eyes open (*F*(2. 42) = 3.41, **p* *= 0.04, p*η*^*2*^* *= 0.140). Pairwise comparisons indicated a significant decrease at mid-test in the PRT group (difference = 67.08, **p* *= 0.02). With eyes closed, pairwise comparisons revealed a significant decrease in MS-X from pretest to mid-test in both the PRT (difference = 65.69, **p* *= 0.005) and PLT groups (difference = 51.25, **p* *= 0.04). Significant time effects on X-Diff were observed in both the PRT (*F*(2, 42) = 11.86, **p* *< 0.001, p*η*^*2*^* *= 0.361) and PLT groups (*F*(1.25, 20) = 13.44, **p* *= 0.00, p*η*^*2*^* *= 0.457). Pairwise comparisons revealed a significant decrease in X-Diff from pre-test to post-test in both the PRT (difference = 47.22, **p* *= 0.006) and PLT groups (difference = 58.22, **p* *< 0.001).

### Sensitivity analysis

Results from the sensitivity test based on the available data were consistent with the from the ITT analysis.

## Discussion

This study examined the effects of Pilates combined with breathing exercise on lung function, body posture, and postural stability in female college students with poor body posture. Compared to Pilate only, PRT showed more positive effects on all test parameters, especially in those lung function related parameters. These concurrent improvements in lung and postural stability may reflect underlying physiological links, particularly involving respiratory muscle function. While the present study did not directly assess interaction, existing evidence suggests a bidirectional relationship between respiratory muscles function and core stability [29–33]. This possibility merits further exploration in future studies.

A significant improvement trend in lung function was observed in both the PRT and PLT groups. In the PLT group, only FEV1% and TV improved significantly at mid-test. In contrast, the PRT group exhibited significant pre-to-post improvements in FEV1%, PEFR, and TV. This indicates the necessity of extending the intervention duration (beyond 16 weeks) to achieve appropriate effects on lung function in the target population. Further studies should explore the dosage relationship between Pilate training and changes in lung function.

Although supervised, group-based exercise interventions are commonly preferred in related studies to ensure exercise quality, the present study discovered an interesting possibility that self-practice may help maintain the persistence of training effects. In the present study, given the forbidden policy during COVID-19, group-based exercise intervention has to be stopped after the mid-test, and the home-based self-practice was conducted afterwards. Based upon the test results at the end of the 16-week intervention, continuous training effects were found on all test parameters. This suggests that exercise for highly educated populations, such as college students, does not have to be limited to group training. Home-based self-practice exercises can also produce significant training effects.

The significant effect of PRT on lung function is mainly due to the addition of breathing exercises to Pilates. Breathing exercises serve as a direct and effective method for training the respiratory muscles. According to the present findings, even 10 weeks of equipment-based Pilates exercises had some positive effects on respiratory parameters. However, its effectiveness was clearly enhanced when combined with breathing exercises. Holding the breath briefly after inhalation increases pressure on the diaphragm by up to 40%, thereby enhancing its strength and improving the overall respiratory process. [34]. There is an obvious increase in respiratory muscle during inspiratory muscle resistance training alone. Such training effects would be enhanced alongside aerobic training [35]. Moreover, the increased respiratory muscle strength and coordination increase the amount of oxygen inhaled into the lungs, ultimately enhancing lung ventilation.

Pilates stretches the auxiliary respiratory muscles (e.g., sternocleidomastoid, trapezius, pectoralis major), and the reduced involvement of the auxiliary muscles in respiratory movements would finally increase abdominal strength and activates the primary respiratory muscles [29]. Improved respiratory efficiency would result in significant improvements in exercise capacity and pulmonary ventilation [30–32]. Interestingly, activation of core muscle strength (including respiratory muscles) can partially enhance postural alignment and increase breathing capacity [33]. Therefore, combining breathing exercises into Pilates sessions may be a simple and effective way to enhance respiratory muscle strength and endurance in female college students with poor posture. Furthermore, the absence of adverse effects and high adherence rates during the intervention in the PRT group also demonstrated its safety and feasibility in the target population. Moreover, the PRT group shows consistent improvement in all primary and secondary variables, suggesting that the changes observed in the PRT group were not due to chance. Taken together, this study suggest that combining Pilates with physiotherapy interventions, such as breathing exercise, could be an effective way of improving poor posture.

Postural stability relies on the coordinated functioning of the visual system, the brain, and the neuromuscular system [36,37]. This study calculated the static postural stability index by analyzing the trajectory of postural sway during a one-legged stance with eyes open or closed. Similar studies have shown that core stability training can significantly improve the static balance in both healthy individuals and those with visual impairments [38]. Incorporating inspiratory muscle resistance training into Pilates may improve the effectiveness of the training and enhance the stability of the deep core muscles [39]. Similar results have been reported in related studies that incorporated respiratory muscle-targeted training into general physical training. The increased diaphragm thickness and diaphragmatic excursion help to explain the shorter X-axis displacements, reduced COP excursions, and improved postural stability [40,41].

## Conclusions

Combining breathing exercises with Pilates can effectively improve lung function, poor body posture, and postural stability in female college students, and a longer training duration (> 16 weeks) would be preferred. These findings raise the possibility of a synergistic relationship between lung function and postural control, potentially mediated by respiratory muscles. However, this hypothesis requires further empirical validation.

### Limitations

This study has several limitations. First, although home-based training sessions were delivered live and supervised in real time via Tencent Meeting, the remote nature of supervision especially during the COVID-19 pandemic may have introduced some variability in exercise execution and adherence compared to in-person monitoring. Second, some postural assessments were conducted using photogrammetry. While this method is practical and non-invasive, it involves a degree of subjectivity that may limit the precision of the measurements.

## Supporting information

S1 File Dataset. (XLSX)

S2 File CONSORT-2010-checklist. (DOC)

S3 File Protocol. (DOCX)
