# Supplementary material for: Effects of Pilates combined with breathing exercise on lung function, body posture and postural stability among female college students: A randomized controlled trial
Source: PLoS One. 2025 Aug 20;20(8):e0330874. doi: 10.1371/journal.pone.0330874 (PMC12367138; doi:10.1371/journal.pone.0330874)
Supplement: S3 File — (DOCX) [file pone.0330874.s003.docx]

Research protocol

Part I: Research Plan

Note: The following research plan was prepared in 2020 and subsequently approved prior to the initiation of the trial by the Ethics Committee of Nanjing Normal university (dated March 2020)

**Project summary**

This is a randomized, single-blinded, parallel-controlled, three-group trial. A total of 66 females (mean age 19 years) with poor body posture were recruited from a local university and randomly divided into three groups, Pilates combined with breathing exercise training (PRT) group, Pilates group (PLT), and control group (CON). Randomized participants in each intervention arm will receive a three weekly, 60-minute exercise program for 16 consecutive weeks. Primary and secondary outcome measures will be collected at baseline, 8 weeks, and 16 weeks. An innovative inference reached that PRT can stimulate the diaphragm and strengthen the deep stabilizing muscles and trunk control, the function of the respiratory system may be improved to a certain extent by exercising the core muscles, including the respiratory muscles. It also indirectly affects muscle balance, resulting in improved static postural stability. Thus, the potential mechanism of linkage mechanism shall exsit between respiratory muscles and postural control during Pilates practice that warrants further study. Based on theoretical considerations and previous research on PRT, the overarching working hypothesis is that PRT will show better performance results on primary outcome measures compared to both the PLT training and CON.

**General information**

- The authors have provided this additional section of the Supplementary Material to furnish readers with further details about the study's protocol, encompassing its design and methodologies.
- The task of gathering body posture data was undertaken by Yaqing Hu, while Hao Dong took charge of measuring static postural control data. Xuejiao Zhou oversaw the experiment's questionnaire, and Huasheng Yao managed lung function data. These researchers, graduate students from Nanjing Normal University's School of Sport Science, possess extensive experience. Tel: 18066089973
- Data will be collected in the physiological laboratory on the first floor of the College of Sports Science at Nanjing Normal University (NJNU).

**Rationale & background information**

Regular exercise behavior is positively related to the health of respiratory function and the development of postural muscle groups^1^. Exercise or sole respiratory muscle training have been demonstrated to be effective in improving dyspnea in patients with the chronic obstructive pulmonary disease^2^. Pilates is an integrated exercise focusing on strength, core stability, flexibility, muscle control, posture, and breathing^3,4^. During exercising Pilates, it allows one to exercise the deep muscle groups that maintain and coordinate body posture while supporting and protecting the spine^6,7,11^. The deep and core muscles, which include the transverse abdominis, multifidus, pelvic floor and diaphragm muscles, can be activated to provide alignment and support for posture^12,13,14^. In addition, these deep and core muscle groups can strengthen small muscles in the abdomen, lower back, hip and spine, which would further balance back and abdominal muscle strength and correct poor body posture^5^. However, a problem exist in the related studies is that few studies have been conducted to examine the effectiveness of exercise can effectively improve abnormal body posture while intervening to improve respiratory function or whether exercise improves respiratory function by targeting abnormal body posture muscle groups. Therefore, this study aimed to examine the effects of exercise on lung function, body posture and postural stability among female college students with poor body posture and the linking effects of an exercise intervention on respiratory and postural stabilizing muscle groups.

**Study goals and objectives**

The primary aim is to investigate whether the PRT program can improve primary outcome measures of lung function and secondary outcome measures of standing posture and static postural stability in female university students. Two secondary aims are to be examined: (a) whether there is a synergistic effect exist in the improvements of lung function and body posture and (b) whether respiratory muscles can mediate synergistic changes in lung function and posture.

**Study design**

We propose to conduct a 4-month randomized, controlled, single-blind trial with three arms. The study will be recruited through posters and presentations on healthy lifestyle promotion, where eligible participants will undergo initial screening, consent and baseline assessments, and will be numbered and randomized into three equal groups. Details regarding study population, subject recruitment, randomization, intervention components, and outcome assessments are described below.

**Study Population**

The intervention will be directed toward individuals who have a poor body posture, with the following eligibility criteria.

Inclusion criteria will include (a) long-term sedentary study work; (b) no regular daily exercise habit; (c) had one of the poor body postures such as rounded shoulders (natural upright posture with strongly forward-rotated upper limbs and raised shoulder blades), forward head posture (forward angle <48°), anterior pelvic tilt (anterior pelvic tilt angle >15°). Participants were excluded if they displayed any physical unfitness for exercise or time conflict with intervention.

**Recruitment Sources and Procedures**

Potential study participants will be recruited through three major sources: (1) recruitment information posted on WeChat (online) circle of friends; (2) poster campaign; (3) lectures about healthy lifestyle promotion. The study participants will be

recruited from the University in Nanjing. To reduce potential expectation bias, participants will be informed that this study will compare three different groups and that participants will be randomly assigned to either group.

Postural assessments will be conducted by researchers with a professional background in sports human science.

**Baseline Assessments**  Those individuals who are eligible after screening will then be given informed consent materials followed by a baseline evaluation conducted by a study assessor. The baseline assessments will include body mass index (BMI), height, bust circumference, waist circumference, hip circumference, and collection of demographics, health status, and medication use. This will be followed by assessments of lung function, and static posture stability. At that time, the intervention activities involved with the study will be reiterated to the eligible individuals. Those who meet all study criteria, are clear about research procedures, have completed baseline assessments, and give final consent will then be randomized.

**Randomization and Blinding**

Eligible participants who have signed the informed consent form will be entered into the computer and divided into three equal groups.

All study assessors who will collect study outcome measures will be blinded to study hypotheses and group allocation. Blinding will be strictly maintained by emphasizing to assessors the importance of minimizing assessment bias and regular checking of the blinding status. Participants will be instructed not to reveal their group status to the study assessors at any time.

**Intervention Instructors**

The PRT was conducted at 16:00-18:00 on Monday, Wednesday and Friday, whereas PLT was conducted at the same time slots on Tuesday, Thursday and Saturday. One qualified Pilate instructor trained the two groups in the same gym hall to ensure that the participants in the two exercise groups received the same training guidance.

**Class Location**

The first eight weeks involved supervised exercise at a gym, followed by eight weeks (weeks 9-16) of home-based online training.

**Intervention Adherence**  Every participant is expected to commit to a four-month period of intervention. Attendees will be motivated to join every session of the intervention (totaling 48), anticipating a compliance rate exceeding 85%. A research assistant will meticulously track class participation every two weeks in the initial month, followed by monthly checks thereafter. Individuals will be classified as drop-outs if they discontinue participation in the intervention, do not come back for assessments, or are absent for over four weeks in a row during their designated intervention. Every participant ceasing participation in the intervention sessions will be promptly reached out to and urged to come back for all assessments. Every participant who dropped out will be subjected to an exit interview to ascertain their reasons for discontinuing the study.

**Drop-outs**

Unavoidable drop-outs, such as scheduling conflict, are anticipated. Based on our prior studies involving Pilates, we estimate a 15% dropout rate for the overall study. The proposed sample size will take into account this anticipated dropout rate.

**Outcome Measure**

All primary and secondary measures will be ascertained at baseline, 8 weeks (midpoint), 16 weeks (intervention termination).

**Study Assessors**

All study assessors will be oriented and trained to follow the general assessment

guidelines and protocols established by the research project. Training will be conducted by the Principal Investigator. Following the established assessment protocol, the assessors will also be trained by a board-certified neurologist to use the Unified Parkinson’s Disease Rating Scale. Inter-rater reliability among assessors will be established via an intraclass correlation method.

**Methodology**

**Outcome measures**

The Lung Function Tester (AS-507, Japan) was employed to evaluate respiratory capacity, following a comprehensive lung function analysis that encompassed forced vital capacity (FVC), forced expiratory volume in the first second (FEV1), FEV1/FVC, peak expiratory flow rate (PEFR), maximum ventilation volume (MV), minute ventilation volume (MV), and tidal volume (TV). The test for respiratory function was performed thrice, with the highest score being documented. Respiratory muscle function was measured using PEFR and MVV, while lung function was assessed using FVC, FEV1%, MV, and TV. The subjects were advised to remain seated for a duration of 10 minutes prior to the examination to prevent any possible respiratory impacts.

Photogrammetry^10^ was employed to evaluate the stationary standing stance. The participants were instructed to maintain a fully relaxed and natural posture, oriented towards a hypothetical point on the wall across. Red patches were used to mark the seventh cervical spinous process, the tip of the shoulder, and the ear screen. Concurrently, the front upper and back upper parts of the iliac spine were identified by a yellow patch. The subjects positioned their left shoulder against the wall, facing the chart for assessing posture. Positioned two meters away from the body assessment chart, the photo tripod holding the digital camera aligned perfectly with the participant's right shoulder. The smaller the data, the better the static postural stability. Side images were captured, and the participant's round shoulder angle (RSA) and anterior pelvic tilt angle (APA) while standing were computed using Photoshop. Manual measurement of the gap between the two shoulder peaks (DSP) and the anterior pelvic tilt dimension (APD) was performed using a soft ruler tape. Positioned parallel to the body's frontal axis, the soft tape gauges the peak points between the left and right shoulders. Additionally, the front part of the pelvic tilt was gauged 2 cm beneath the participant's navel.

Participant characteristics. Demographic and health characteristics of participants will be collected at baseline to describe the sample, compare conditions, and investigate characteristics associated with outcomes. These measures will include age, gender, use of medication, resting blood pressure, body weight (kg), and body height (cm), Bust Circumference (cm), Waist Circumference (cm), Hip circumference (cm). Blood pressure will be measured with the use of an automated device (Omron HealthCare). Body weight and height will be assessed through the use of digital scales (Health o Meter@).

**Testing Procedures**

The assessment protocol will be standardized to ensure measurement consistency between assessors and across different sites and to minimize effects of motor fluctuations. Efforts will be made to schedule all assessments at the same time of day and to perform them in the same.

**Safety considerations**

Adverse events, major or minor, during a class session or outside classes (school, lab testing) will be documented in a project adverse log and, in the event of observing a major adverse event, reported immediately to the Ethics Committee of Nanjing Normal University.

In-class exercise safety will be closely monitored during the entire length of the trial by research staff and class instructors. Staff will make periodic visits to classes to check safety status and address concerns related to the exercise programs. Intervention instructors will be asked to monitor participants for symptoms of any discomfort. Modifications in the training protocol will be made, upon approval of the research staff, on an individual basis as necessary.

**Follow-up**

Ongoing attention to participants' posture and exercise adherence after the exercise intervention has ended. A follow-up phone call is made to check the physical status.

**Data management and statistical analysis**

**Preliminary analysis**. Before addressing the main questions of the study, we will examine whether attrition influenced the representativeness of the remaining study sample, whether the intervention conditions were different on demographic variables that are not controlled for in the random assignment procedure, and whether any of the baseline characteristics (e.g., age) need to be accounted for in the primary analyses. Group comparisons on baseline demographic descriptors and primary and secondary outcome measures will be performed using analysis of variance for continuous variables and the chi-square (or Fisher’s Exact) test for categorical variables.

**Main analysis.** To evaluate the effects of PRT on the a priori specified outcomes, a repeated-measures, which will include all assigned participants consistent with the principle of intention-to-treat. The dependent variables will be measures of primary and secondary outcomes collected at baseline, 8 weeks, and 16 weeks, operationalized as continuous variables. Independent variables will be restricted to basic design features: fixed effects for treatment group and time. A one-way analysis of covariance (ANCOVA) taking baseline as the covariance was performed at mid-test and post-test to determine group effects on each outcome parameter.

**Quality assurance**

Implementation will include a uniform intervention protocol and a checklist for process evaluation, formulated from previous trials^1,3^. The primary emphasis of these strategies is on maintaining the integrity of the intervention, encompassing aspects like (a) the training and qualification of instructors, (b) the allocation of various forms or movements in teaching, (d) the uniformity and intensity of exercise dosages at different locations, and (e) regular weekly checks and monitoring of class attendance. Authorized personnel will carry out the assessment monthly.

**Expected outcomes of the study**

Exercise will be effective in intervening to improve respiratory function while improving abnormal body posture, or exercise will improve respiratory function by targeting abnormal body muscle groups. This result is very important for correcting poor posture in this population. It is important to focus not only on the intervention of the postural muscle groups, but also on the respiratory muscle groups during the correction of body posture.

**Dissemination of results and publication policy**

Ensuring that the results of the experiment will be disseminated not only in the scientific media for results, but also to the participants, will be recognised by sedentary people, especially those with poor body posture.

**Duration of the project**

**Interventions.** The protocol will ensure that each exercise group has a consistent intervention schedule on key training parameters involving duration (60 minutes per session), frequency (3 times per week), and training structure (i,e., a 5-minute warm-up, Pilates training, a 10-minute breathing trainer training PRT group only, and a 5-minute cool-down). Participants in all two conditions will be instructed not to engage in any additional home practice.

**Pilates.** The protocol consists of 24 Pilates movements^8^. The names of 24 movements are as follows: drawing circles with hands, march stepping, four-legged swimming, curl-ups, 100 taps, plank, chest lift, single-leg drawing circles, quadriceps stretch, opposite arm and leg reach, supine spinal rotation, prone props, prone swimming, swan, v-spins, con-con dance, double leg extensions, mermaid side bends, side lying pedal cycle, shoulder bridge, prone swimming, quadriceps stretch, kneeling side kick, neck and shoulder stretch.

The 16-week exercise program was divided into four stages (ice breaker, for beginners, for intermediate, and for advanced), and the quality of movements and exercise intensity were gradually increased along with training process. Because the goal of the protocol is to train participants' in core muscles and correct poor posture, the protocol is specifically designed to challenge core muscle control and spinal flexibility and functional training modalities. Instruction will cover learning new forms and reviewing and practicing forms learned in previous sessions.

At the end of each phase, self-perceived exercise intensity was assessed using the Chinese version of the Rating of Perceived Exertion (RPE) scale^9^. Exercise intensities were ranged from light (RPE 11) to lightly hard (RPE 13). The exercise load was arranged 3-4 sets per group and 6-9 repetitions per set. Exercise intensity as measured by heart rate were recorded before, during and after each training session.

**Breathing exercise.** In the PRT group, participants were required to use a breathing trainer (TRI-BALL breath-trainer) to perform respiratory muscle exercises (10 minutes) immediately after the Pilates training. Participants were instructed to perform fast and forceful inspirations to achieve maximal inhalation and exhalation during every breath.

**Problems anticipated**

In the event of a sudden outbreak of a novel coronavirus, exercise interventions will be conducted in an online manner to ensure adequate time for exercise interventions.

**Project management**

Author Jie Zhang and author Yanan Zhao have given substantial contributions to the conception or the design of the manuscript, author Jie Zhang and author Qianwen Wang to acquisition, analysis and interpretation of the data. All authors have participated to drafting the manuscript, author Yanan Zhao revised it critically.

**Ethics**

University students served as the study's participants, who experienced bodily alterations during the exercise program, necessitating the application for biomedical ethics to safeguard their legal rights, interests, and safety. Before initiating the exercise program, participants were queried about the suitability of the exercise program, and those agreeing to join the exercise program provided their informed consent.

The duration of this study spans 16 weeks; a set of students will engage in Pilates with respiratory muscle exercises thrice weekly, while another set will partake in Pilates training merely thrice weekly. Concurrently, the team will gather these details from you: 1. fundamental data (name, age, height, etc.) 2. Insights into a healthy way of living (such as exercise, sleep quality, mental well-being) 3. lung function evaluation 4. body posture analysis 5. body composition examination, among other aspects. Tests for Items 2-5 will be conducted thrice, encompassing the periods before, during, and following the formal instruction. Collaborate with the staff to organize the tests sequentially.

1. León-Morillas F, Lozano-Quijada C, Lérida-Ortega M, et al. Relationship between Respiratory Muscle Function and Postural Stability in Male Soccer Players: A Case-Control Study. *Healthcare (Basel)*. **9** (6) (2021).
2. Jimborean G, Ianosi E, Croitoru A, et al. Respiratory muscle training in chronic obstructive pulmonary disease. *Pneumologia*. **66,** 128-130 (2017).
3. Chen H, Li P, Li N, et al. Rehabilitation effects of land and water-based aerobic exercise on lung function, dyspnea, and exercise capacity in patients with chronic obstructive pulmonary disease: A systematic review and meta-analysis. *Medicine (Baltimore)*. **100** (33)**,** e26976 (2021).
4. Song BH, Kim J. Effects of Pilates on Pain, Physical Function, Sleep Quality, and Psychological Factors in Young Women with Dysmenorrhea: A Preliminary Randomized Controlled Study. *Healthcare (Basel)*. **11** (14) (2023).
5. Wells C, Kolt GS, Bialocerkowski A. Defining Pilates exercise: a systematic review. *Complement Ther Med*. **20** (4)**,** 253-262 (2012).
6. Hellyer NJ, Folsom IA, Gaz DV, et al. Respiratory Muscle Activity During Simultaneous Stationary Cycling and Inspiratory Muscle Training. *J Strength Cond Res*. **29** (12)**,** 3517-3522 (2015).
7. van Kleef ESB, van Doorn JLM, Gaytant MA, et al. Respiratory muscle function in patients with nemaline myopathy. *Neuromuscul Disord.* **32** (8)**,** 654-663 (2022).
8. Wu Z: Pilates. Beijing Sports University Press, CHINA (2016).
9. Ding W, You T, Gona PN, et al. Validity and reliability of a Chinese rating of perceived exertion scale in young Mandarin speaking adults. *Sports Med Health Sci.* **2** (3)**,** 153-158 (2020).
10. Singla D, Veqar Z, Hussain ME. Photogrammetric Assessment of Upper Body Posture Using Postural Angles: A Literature Review. J Chiropr Med. 16 (2), 131-138 (2017).
11. van Kleef ESB, van Doorn JLM, Gaytant MA, et al. Respiratory muscle function in patients with nemaline myopathy. *Neuromuscul Disord.* **32** (8)**,** 654-663 (2022).
12. Figueiredo RIN, Azambuja AM, Cureau FV, et al. Inspiratory Muscle Training in COPD. *Respir Care*. **65** (8)**,** 1189-1201 (2020).
13. Tout R, Tayara L, Halimi M. The effects of respiratory muscle training on improvement of the internal and external thoraco-pulmonary respiratory mechanism in COPD patients. *Ann Phys Rehabil Med*. **56** (3)**,** 193-211 (2013).
14. Seo DK, Kim JS, Lee DY, et al. The relationship of abdominal muscles balance and body balance. *J Phys Ther Sci*. **25** (7)**,** 765-767 (2013).

**PartⅡ: Research Plan**

**Budget**

Costs used in the study will be supported by the subject group.

**Other support for the project**

None

**Curriculum Vitae of investigators**

Jie Zhang (1995-), Female, Master of physical education, Nanjing Normal University, China. Physical Education and Training, exercise and health promotion; Teacher, College of physical education, China Three Gorges University, [zhangjie@ctgu.edu.cn](mailto:zhangjie@ctgu.edu.cn).

Qianwen Wang (1993-), Female, Ph.D., The department of Orthopedics & Traumatology, The Chinese University of Hong Kong, Hong Kong ,999077,China. [alicia157@link.cuhk.edu.hk](mailto:alicia157@link.cuhk.edu.hk).

Yanan Zhao* (1987-), Female, Professor, Ph.D., Postgraduate, School of Sports Science and Physical Education, Nanjing Normal University, Sport Science, exercise and health promotion; 025-85891407, [ynzhao@njnu.edu.cn](mailto:ynzhao@njnu.edu.cn).

**Authoritative international academic journal articles（SCI/SSCI）**
**Zhao, Y.,** & Chung, P. K. (2017). Neighborhood Environment Walkability and Health-related Quality of Life among Older Adults in Hong Kong. *Archives of Gerontology and Geriatrics* (SSCI /SCIE; 2016 IF =2.086), 73(6), 182-186.doi: 10.1016/j.archger.2017.08.003.
**Zhao, Y. N.**, Chung, P. K, & T.K. Tong (2017) Effectiveness of a balance-focused exercise program for enhancing functional fitness of older adults at risk of falling: a randomized, controlled study. *Journal of Geriatric Nursing* (SSCI/SCIE, 2016 IF = 1.142). 38(6), 491-497. doi: 10.1016/j.gerinurse.2017.02.011
Chung, P. K., **Zhao, Y. N**., Liu, J. D., & Quach, B. (2017) A Canonical correlation analysis of functional fitness and quality of life in older population. *Archives of Gerontology and Geriatrics* (SSCI/SCIE; 2016 IF =2.086). 68（1）,44-48 doi: 10.1016/j.archger.2016.08.007.
**Zhao, Y. N.**, Chung, P. K, & Tomas K. TONG (2016) Effectiveness of a community-based exercise program on balance performance and fear of falling in older nonfallers at risk of falling: a randomized, controlled study. *Journal of Aging and Physical Activity* (SSCI/SCI; 2015 IF = 1.873). 24（4），516-524
Chung, P. K., **Zhao, Y. N.**, Liu, J. D., & Quach, B. (2016) Functional Fitness Norms for Community-dwelling Older Adults in Hong Kong. *Archives of Gerontology and Geriatrics* (SSCI /SCIE; 2015 IF = 1.971). 65（4）,54-62.
**Zhao, Y. N.** & Chung, P. K. (2016) Differences of Functional Fitness between Older Adults with and without Risk of Falls. *Asian Nursing Research* (SSCI/ SCIE; 2015 IF = 1.000). Asian Nursing Research, 10(1), 51-55.
Chung, P. K., **Zhao, Y. N.,** Liu, J. D., & Quach, B. (2015) Validity and Reliability of the Rating of Perceived Scale in Monitoring Exercise Intensity among Chinese Older Adults in Hong Kong. *Perceptual & Motor Skill* (SSCI; 2014 IF = 0.546). 121(3), 805-809.

Zhu, C., Du, Y., & **Zhao, Y***. (2023.1). Subjective and Objective Physical Activity Measurement Methods for the Prediction of Possible Sarcopenia. Med Discoveries, 2(3), 1021.

Liu D, Zhang Y, Wu L, Guo J, Yu X, Yao H, Han R, Ma T, Zheng Y, Gao Q, Fang Q, Zhao Y, **Zhao Y***, Sun B, Jia W and Li H (July, 2022) Effects of Exercise Intervention on Type 2 Diabetes Patients With Abdominal Obesity and Low Thigh Circumference (EXTEND): Study Protocol for a Randomized Controlled Trial. Front. Endocrinol. 13:937264.

Wang, H., Huang, Y., **Zhao, Y.*** (July, 2022) Efficacy of Exercise on Muscle Function and Physical Performance in Older Adults with Sarcopenia: An Updated Systematic Review and Meta-Analysis. Int. J. Environ. Res. Public Health, 19, 8212.

**Zhao, Y**.*, Wang, Z., Chung, P. K., & Wang, S. (Sep.2021). Functional fitness norms and trends of community-dwelling older adults in urban China. Scientific Reports, 11(1), 1-8.

**Zhao Y***, Cai K, Wang Q, Hu Y, Wei L, Gao H. (March, 2021) Effect of Tap dance on plantar pressure, postural stability and lower body functions in diabetic patients at risk of diabetic foot: a randomized controlled trial. BMJ Open Diabetes Research & Care.

Wu, T & **Zhao, Y^*^.** (March–April, 2021). Associations between functional fitness and walking speed in older adults. Geriatric Nursing, 42(2), 540-543.

**Zhao, Y*.**, Wang, Q., Pak-Kwong, C., & Cai, K. (January, 2021). Cross-cultural modifications and measurement properties of the CHAMPS questionnaire among Chinese older adults. Journal of Exercise Science & Fitness, 19(1), 13-18.

**Zhao, Y*.**, Wu, T., & Wei, Y. (May, 2020). Effects of starting position, distance and ending point in a walking speed test among older adults. Geriatrics & Gerontology International, 20(7), 680-684.

Wang, Q., **Zhao, Y^*^**.(July, 2021) Effects of a Modified Tap Dance Program on Ankle Function and Postural Control in Older Adults: A Randomized Controlled Trial. Int. J. Environ. Res. Public Health, 18, 6379.

Chung, P. K., Zhang, C. Q., **Zhao, Y.**, Wong, M. Y. C., & Hu, C. (2021). Effectiveness of resistance training on resilience in Hong Kong Chinese older adults: study protocol for a randomized controlled trial. BMC geriatrics, 21(1), 1-8

Chu Y, **Zhao Y**, Hu S, Wang Q, Semeah LM, Jia H, Lv T, Li X, Wang R. (August, 2020) Immediate Effect of Local Vibration Therapy for Sport-induced Fatigue Based on Traditional Chinese Medicine's Holistic Theory. Journal of Multidisciplinary Healthcare.
